# *CASQ2* variants in Chinese children with catecholaminergic polymorphic ventricular tachycardia

**DOI:** 10.1002/mgg3.949

**Published:** 2019-09-03

**Authors:** Qirui Li, Ruolan Guo, Lu Gao, Lang Cui, Zhihui Zhao, Xia Yu, Yue Yuan, Xiwei Xu

**Affiliations:** ^1^ Department of Cardiology Beijing Children’s Hospital Capital Medical University National Center for Children’s Health Beijing China; ^2^ Beijing Key Laboratory for Genetics of Birth Defects Beijing Pediatric Research Institute Beijing China; ^3^ Genetics and Birth Defects Control Center National Center for Children's Health Beijing China; ^4^ MOE Key Laboratory of Major Diseases in Children Beijing China; ^5^ Beijing Children's Hospital Capital Medical University Beijing China; ^6^ Internal Medicine Teaching and Research Department Beijing Children’s Hospital Capital Medical University National Center for Children’s Health Beijing China

**Keywords:** autosomal recessive, *CASQ2* variants, catecholaminergic polymorphic ventricular tachycardia, targeted next‐generation sequencing

## Abstract

**Background:**

Biallelic variants of the *CASQ2* are known to cause the autosomal recessive form of catecholaminergic polymorphic ventricular tachycardia (CPVT), an inherited disease that predisposes young individuals to syncope and sudden cardiac death. To date, only about 24 *CASQ2* variants have been reported in association with CPVT pathogenesis; furthermore, studies in Asians, especially in the Chinese population, are relatively rare. The aim of this study was to detect *CASQ2* variants in Chinese patients with CPVT.

**Methods:**

We used targeted next‐generation sequencing (NGS) to identify *CASQ2* variants in Chinese patients with CPVT. A screening process was performed to prioritize rare variants of potential functional significance. Sanger sequencing was conducted to conform the candidate variants and determine the parental origin.

**Results:**

We identified seven different *CASQ2* variants, of which three (c.1074_1075delinsC, c.1175_1178delACAG, and c.838+1G>A) have not been previously reported. The variants exhibited autosomal recessive inheritance, and were detected in four unrelated Chinese families with CPVT. They included a nonsense variant c.97C>T (p.R33*) and a missense variant c.748C>T (p.R250C) in Family 1 with three CPVT patients; two heterozygous frameshift variants, c.1074_1075delinsC (p.G359Afs*12) and c.1175_1178delACAG (p.D392Vfs*84), in Family 2 with one CPVT patient; one pathogenic homozygous variant c.98G>A (p.R33Q) of *CASQ2* in the CPVT patient of Family 3; and two heterozygous splicing variants, (c.532+1G>A) and (c.838+1G>A), in Family 4 with one CPVT patient.

**Conclusion:**

To our knowledge, this is the first systematic study of Chinese children with *CASQ2* variants. Our work further expands the genetic spectrum of *CASQ2*‐associated CPVT.

## 1 INTRODUCTION

Catecholaminergic polymorphic ventricular tachycardia (CPVT) is a rare but severe, inherited cardiac arrhythmic disorder characterized by bidirectional and/or polymorphic ventricular tachycardia (bVT/pVT), syncope, and cardiac arrest during exercise or emotional stress in children and young adults without structural heart disease and with normal baseline electrocardiogram (Pflaumer & Davis, 2019). CPVT is one of the most common causes of syncope and sudden death in both adolescents and children (Vacanti, Maragna, Priori, & Mazzanti, 2017). Patients with CPVT typically present for medical attention most frequently during childhood; the mean age at first symptom varies from 7 to 9 years, although early onset has also been reported (Landstrom et al., 2017; Roston, Haji‐Ghassemi, et al., 2018; Roston, Yuchi, et al., 2018). Despite these presumptive symptoms, because of the lack of knowledge and inadequate recognition of CPVT, delayed or missed diagnosis of CPVT in children is common (Jiang et al., 2018; Roston et al., 2015). If left untreated, the mortality rate of symptomatic CPVT patients below the age of 30 years is 31% (Tan, Hofman, van Langen, van der Wal, & Wilde, 2005).

Variants in genes that regulate cellular calcium homeostasis are the main contributors to CPVT, which is characterized by excessive calcium leakage from the sarcoplasmic reticulum, leading to delayed afterdepolarizations and arrhythmias (Faggioni & Knollmann, 2012). The majority of CPVT cases are usually caused by heterozygous variants in the gene encoding cardiac ryanodine receptor‐2 (*RyR2*, OMIM: 180902) (Laitinen et al., 2001) on chromosome 1q43. The second most common type of CPVT is caused by homozygous or compound heterozygous variants in the gene encoding cardiac calsequestrin (*CASQ2*, OMIM: 114251) on chromosome 1p13 (Lahat et al., 2001). Although less recognized, *SCN5A* (OMIM: 600163), *TRDN* (OMIM: 603283), *CALM1* (OMIM: 114180), *CALM2* (OMIM: 114182), and *CALM3* (OMIM: 114183) have also been implicated in CPVT (Paludan‐Müller et al., 2017; Roston, Haji‐Ghassemi, et al., 2018; Roston, Yuchi, et al., 2018). However, patients with *CASQ2* variants have earlier ages of onset, more serious clinical presentation, and higher mortality rates when untreated than that observed in patients with *RyR2* variants (Sumitomo, 2016). Therefore, screening and analysis of *CASQ2* variants are of significant clinical value.

The *CASQ2*, which was mapped on chromosome 1p13, contains 2,528 nucleotides and 11 exons (GenBank transcript ID: NM_001232.3, Ensembl transcript ID: ENST00000261448). The CASQ2 protein is a precursor protein of 399 amino acids, which serves as the major calcium ion reservoir within the sarcoplasmic reticulum of cardiac myocytes and is part of a protein complex that contains the ryanodine receptor. In 2001, Lahat et al. first identified a missense variant in a highly conserved region of *CASQ2* associated with autosomal recessive CPVT (Lahat et al., 2001). To date, only 24 variants in the *CASQ2* have been reported to be disease‐causing mutation associated with CPVT, according to the Human Genetics Mutations Database (HGMD Professional 2019.1, http://www.hgmd.cf.ac.uk/ac/index.php). Cardiac calsequestrin, which is the most abundant Ca^2+^‐binding protein in the junctional sarcoplasmic reticulum, plays a critical role in maintaining cardiac Ca^2+^ homeostasis necessary for excitation–contraction coupling in working cardiomyocytes (Kurtzwald‐Josefson et al., 2017). Thus, to expand the variant spectrum of *CASQ2*‐related CPVT, we analyzed the clinical features and gene characteristics of six Chinese children with *CASQ2* variants in this study.

## 2 MATERIALS AND METHODS

### 2.1 Patients

Six Chinese children with *CASQ2* variants from four different non‐consanguineous families were clinically diagnosed with CPVT at the Department of Cardiology of Beijing Children's Hospital (National Center for Children's Health) from January 2017 to December 2017. The diagnosis of CPVT was based on the presence of stress‐induced reproducible bVT or pVT in patients with a normal resting electrocardiogram and no detectable structural heart abnormalities, in accordance with previously published criteria (Priori et al., 2013). We excluded patients who suffered bVT or pVT due to prominent electrolyte imbalance, drug treatment, organic heart diseases, and noncardiogenic entities such as epilepsy or vasovagal syncope. The study was performed in accordance with the Declaration of Helsinki and approved by the Ethics Committee of Beijing Children's Hospital, China (No: 2018‐k‐121). Family members of patients voluntarily provided informed consent.

### 2.2 Genomic DNA extraction

At least 2 ml of peripheral venous blood (in EDTA anticoagulant) was collected from the patients. Genomic DNA was extracted using the standard phenol–chloroform method.

### 2.3 Targeted next‐generation sequencing and analyses

SeqCapEZ Choice XL Library Kits (Roche Nimble Gen) were used to capture the coding exons and intron–exon junctions of all known CPVT‐associated genes, including *RyR2*, *CASQ2*, *TRDN*, *CALM1‐3*, *TECRL*, *ANK2*, and *KCNJ2*. Next‐generation sequencing (NGS) was performed on the HiSeq 4000 Sequencing System. The overall sequencing coverage of the target regions was 98.95% for 200 × depth of coverage in each of the genes. Raw sequencing reads were converted into FASTQ format and mapped against the Human Genome Assembly GRCh37/hg19. Single‐nucleotide variants and indels were called using the *SAMtools* pipeline and annotated using ANNOVAR. Variant frequency was analyzed using four SNP databases (db‐SNP 147, gnomAD, ExAC, and 1000 Genomes Project Database) and two disease databases (ClinVar and HGMD). We filtered all common variants with minor allele frequency >0.05. For the candidate gene variants, we used the following four applications to predict their potential impact on protein function: SIFT (http://sift.jcvi.org/www/SIFT_enst_submit.html), Polyphen‐2(http://genetics.bwh.harvard.edu/pph2/), Mutation Taster (http://www.mutationtaster.org/), and CADD (http://cadd.gs.washington.edu/). Loss‐of‐function variants, such as nonsense/splicing variants and frameshift indels, were considered deleterious. All candidate variants were classified and interpreted according to the American College of Medical Genetics and Genomics (ACMG) criteria (Richards et al., 2015).

### 2.4 Sanger sequencing

PCR amplification and Sanger sequencing were used to confirm the causative variants detected by NGS. PCR products were analyzed on an automatic DNA Sequencer (ABI 3130; Applied Biosystems) using standard methods. Subsequently, the variants identified in the patients were screened in all available family members to determine whether the variants co‐segregated with the disease in the family.

## 3 RESULTS

### 3.1 Clinical features of patients harboring variants

**Figure 1:**
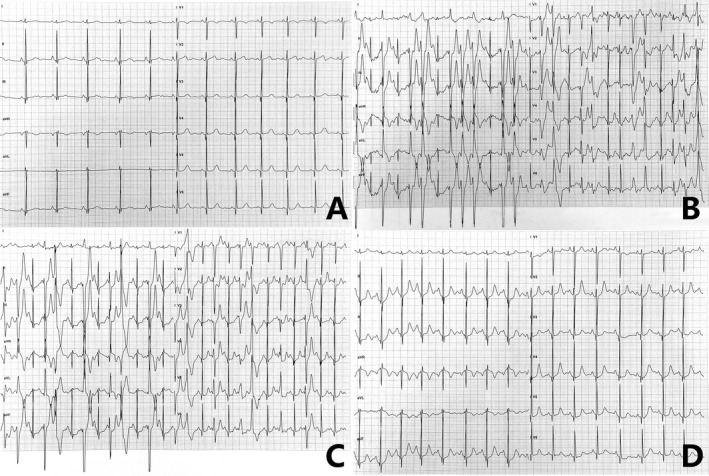
Electrocardiographic manifestations on exercise testing for patient 6. (A) normal 12‐lead baseline electrocardiogram before exercise; (B and C) bidirectional ventricular tachycardia and polymorphic ventricular tachycardia induced by exercise; (D) restoration of normal sinus rhythm after exercise

Patients in all four families shared typical features of CPVT. Family histories were negative for sudden death. In Family 1, before the availability of genetic testing, initial family clinical screening showed a younger brother (of patient 2) had experienced syncope only once during exercise at 8 years old, while both parents and the remaining three siblings were normal. However, during follow‐up, the youngest brother (of patient 3) developed the same symptoms and was diagnosed with CPVT at the age of 7.7 years. The clinical characteristics of the six patients with *CASQ2* variants are summarized in Table 1 and the exercise testing results for patient 6, which are similar in six patients, are showed in Figure 1. All patients were of Han Chinese ethnicity with onset age of 6.2 ± 1.3 years. The age at diagnosis was 9.6 ± 1.9 years, and time interval from symptom occurrence to diagnosis was 3.4 ± 2.4 years. The main symptom was syncope. The frequency of syncope was from once per year to 3–4 times per month, with the syncope duration ranging from several seconds to a few minutes. All patients were able to regain consciousness spontaneously. Three patients were diagnosed at other hospitals as having epilepsy and did not respond to antiepileptic therapy. In patient 4, the frequency of syncope increased from 1–2 times per year at onset to 3–4 times per month despite taking two types of antiepileptic drugs before CPVT diagnosis. None of the six patients showed significant abnormality on resting 12‐lead electrocardiogram, except patient 5 who showed sinus bradycardia with a slow sinus heart rate of 54 beats/min. All patients had positive stress test results (exercise test or Holter) for bVT or pVT.

**Table 1:** Clinical characteristics and treatment of CPVT patients

Family number	Family 1			Family 2	Family 3	Family 4
Patient number	Patient 1	Patient 2	Patient 3	Patient 4	Patient 5	Patient 6
Gender	F	M	M	F	M	F
Age at onset (years)	5.0	8.0	7.7	4.5	7.0	6.0
Age of diagnosis (years)	12.5	10.3	7.7	7.3	10.1	9.9
Syncope	+	+	+	+	+	+
Inducing factors	Exercise	Exercise	Exercise	Exercise/emotional stress	Emotional stress	Exercise
Frequency	5–6/year	Once	Once	3–4/month	1–2/year	2/year
Diagnosis in other hospitals	Epilepsy	−	−	Epilepsy	Cardiac syncope	Epilepsy
Resting electrocardiogram	Sinus rhythm	Sinus rhythm	Sinus rhythm	Sinus rhythm	Sinus bradycardia	Sinus rhythm
Holter	bVT	bVT&pVT;	−	pVT	bVT&pVT	pVT
Treadmill exercise test	bVT&pVT	bVT&pVT	bVT&pVT	pVT	−	pVT
Treatment (mg/kg.d)	Metoprolol (2.0)	Metoprolol (1.8)	Metoprolol (1.5)	Propranolol (1.0)	Propranolol (2.0)	Propranolol (2.2)
Follow‐up results	Well	Well	Well	Well	ICD	Well
Follow‐up time/months	16	14	14	22	18	15

Abbreviations: bVT, bidirectional ventricular tachycardia; F, female; ICD, implantable cardioverter defibrillation; M, male; pVT, polymorphic ventricular tachycardia; +, positive; −, not do.

### 3.2 Identification of *CASQ2* variants

**Figure 2:**
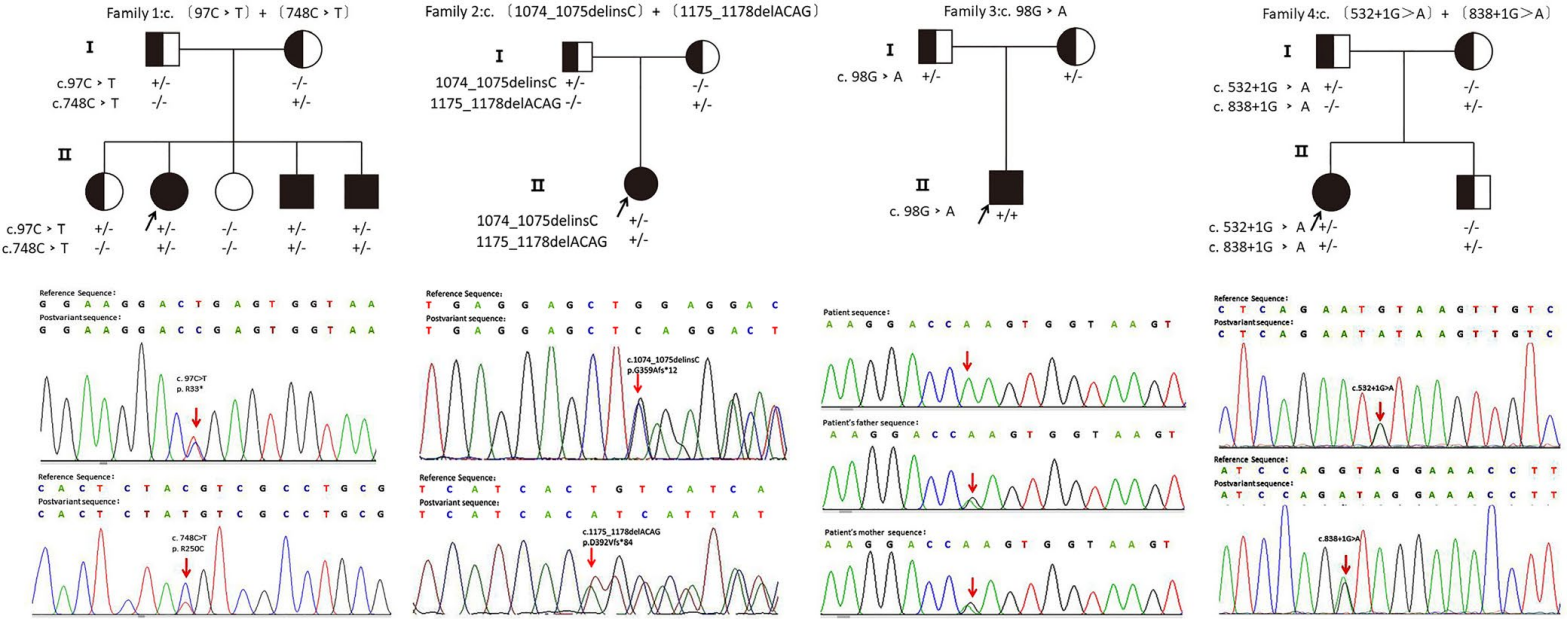
Pedigrees of the four *CASQ2*‐associated CPVT families and variant analysis. Black symbols indicated affected individuals. Half‐filled symbols indicated heterozygous individuals with a single variant. Blank symbols indicated healthy individuals. Black arrows indicated the family probands. Red arrows indicated variants in Sanger sequencing. CPVT, catecholaminergic polymorphic ventricular tachycardia

In our research, seven different *CASQ2* variants were detected in six CPVT patients from four unrelated families. Besides, no variants in *RyR2* nor other genes associated with CPVT were found. As shown in Figure 2, all patients carried homozygous or compound heterozygous variants in *CASQ2* which is consistent with autosome recessive inheritance.

In Family 1, three patients (number 1, 2, and 3) carried the same compound heterozygous variants in *CASQ2*: c.97C>T in exon 1 leading to a nonsense variation that results in an RNA making the truncated protein (p.R33*), and a missense variation c.748C>T in exon 7 resulting in a change from arginine to cysteine at the 250th amino acid (p.R250C). Results of Sanger sequencing for the patients' parents showed that c.97C>T (p.R33*) was inherited from the father, while c.748C>T (p.R250C) was detected in their asymptomatic mother.

Two heterozygous variants (c.1074_1075delinsC and c.1175_1178delACAG) were identified in patient 4 from Family 2. Both variants generated frameshift of the reading frame in exon 11. The variation c.1074_1075delinsC would result in premature occurrence of termination codon (p.G359Afs*12) which was predicted to produce a truncated protein. Another variation c.1175_1178delACAG might cause a termination codon at the 84th base after the variant site, which was predicted to lead to elongation of the protein (p.D392Vfs*84).

A homozygous variant of *CASQ2*: c.98G>A in exon 1 was detected in patient 5 from Family 3. c.98G>A would cause a nonconservative substitution of glutamine for arginine at the 33th amino acid (p.R33Q). Genetic analysis of the parents' DNA revealed that both of them carried a heterozygous variant R33Q.

Compound heterozygous variants c.532+1G>A and c.838+1G>A were identified in patient 6 from Family 4. These two variants located in the conservative donor splicing site of intron 5 and intron 9, respectively. The parents of patient 6, as well as this patient's younger brother, were asymptomatic heterozygous carriers.

**Figure 3:**
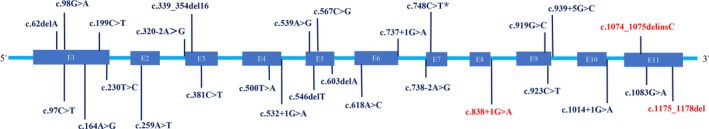
Location of the unreported and known variants in CASQ2. Lines indicate the sites of variants of *CASQ2* on the exons or exon–intron junctions. Blue boxes indicate the 11 exons of *CASQ2*. The unreported and known *CASQ2* variants identified in this study are indicated in red and blue, respectively. *this variant is reported previously in our team but not found in HGMD. HGMD, Human Genetics Mutations Database

By searching literature and databases (HGMD and ClinVar), three of seven different variants we identified were unreported before, including two frameshift variants, c.1074_1075delinsC, c.1175_1178delACAG, and a splicing variant, c.838+1G>A. Other four variants, c.97C>T, c.98G>A, c.748C>T, and c.532+1G>A in *CASQ2*, could be found in HGMD. Details of the gene variants are listed in Table 2 and Figure 3.

**Table 2:** Detailed information of seven variants of *CASQ2* (NM_001232.3) detected in our research

No.	Variant	Chromosome Position (hg19)	gnomAD	SIFT	PolyPhen‐2	Mutation Taster	CADD	NNSPLICE
1	c.97C>T	chr1:116311066	All: 0.000003980 East Asian: 0	NA	NA	Disease causing	Damaging score: 38	NA
2	c.748C>T	chr1:116268164	All: 0.00006582 East Asian:0.00005092	Damaging	Probably damaging	Disease causing	Damaging score: 35	NA
3	c.1074_1075delinsC	chr1:116243987‐116243988	NA	NA	NA	Disease causing	NA	NA
4	c.1175_1178delACAG	chr1:116243885‐116243888	NA	NA	NA	Disease causing	NA	NA
5	c.98G>A	chr1:116311065	All: 0.00001194 East Asian: 0	Damaging	Probably damaging	Disease causing	Damaging score: 35	NA
6	c.532+1G>A	chr1:116280844	NA	NA	NA	NA	NA	Donor site score: decreased
7	c.838+1G>A	chr1:116260460	NA	NA	NA	NA	NA	Donor site score: decreased

### 3.3 Treatment and follow‐up

All six patients were strongly advised to avoid strenuous physical activity and given long‐term oral beta‐blocker therapy. A summary of the treatments is shown in Table 1. Five patients became syncope‐free and asymptomatic during the follow‐up period of 14–22 months. Patient 4 only took a low dosage of propranolol. Implantable cardioverter defibrillation was performed in one patient (patient 5) owing to significant resting bradycardia of 40 beats/min.

## 4 DISCUSSION

In this report, we describe an autosomal recessive form of CPVT that is caused by homozygous or compound heterozygous variants in the *CASQ2*. To our knowledge, this is the first systematic study of *CASQ2* variants in Chinese children, which expands the genetic spectrum of *CASQ2*‐associated CPVT. In our cohort, we identified three unreported variants and four known variants in *CASQ2* (Figure 3). According to the ACMG classification criteria (Richards et al., 2015), c.838+1G＞A was pathogenic (PVS1+PM2+PM3+PP4), while c.1074_1075delinsC (p.G359Afs*12, PM2+PM4+PP4) and c.1175_1178delACAG (p.D392Vfs*84, PM2+PM4+PP4) were considered as variants with uncertain significance.

*CASQ2*‐related recessive variants of CPVT are relatively uncommon, and 10 missense variants, three nonsense variants, one synonymous variant, six splice site variants, and four deletions have been recorded according to the HGMD (updated Professional 2019.1). We recently identified the variant c.748C>T (p.R250C) in Family 1 and predicted that the variant c.748C>T (p.R250C) may change the secondary structure of the original calsequestrin protein: an α‐helix was generated near the 260th amino acid in the original structure, which is not present in the secondary structure of the polypeptide chain in the mutant protein (Gao et al., 2018). However, only few missense *CASQ2* variants have been functionally characterized in vitro. These studies have shown that the variants exhibit impaired Ca^2+^ binding and have the ability to regulate RyR2 channel owing to conformational changes relative to the native calsequestrin protein (Bal et al., 2011; Eckey et al., 2010).

The frameshift variants c.1074_1075delinsC and c.1175_1178delACAG of exon 11 are novel variants that have not been previously reported. The *CASQ2* c.1074_1075delinsC variant is predicted to result in a premature stop codon (p.G359Afs*12). To our knowledge, only one variant of exon 11 in *CASQ2* has been reported as a pathogenic variant for CPVT to date: c.1083G>A, in which a single G to A nucleotide substitution at position 1,083 leads to the formation of a truncated protein (p.W361*) relative to the normal 399‐amino acid calsequestrin protein (Kawamura et al., 2013). The present novel variant of c.1074_1075delinsC is in the upstream of position 1,083 and also produces a truncated protein. Because it is located in the last exon of the gene, therefore, we consider it likely a variant with uncertain significance. Similarly, c.1175_1178delACAG of exon 11 was at the 22nd base, which was located at the C‐terminal region of *CASQ2* and suspected to result in amino acid changes from position 392 (p.D392Vfs*84), producing an elongated protein. Recently, a homozygous p.W361* variant in the last exon of *CASQ2* was identified in a 62‐year‐old female, patient with CPVT onset since childhood for whom sudden death has not been reported to date (Fujisawa et al., 2019). Our results constitute the first report of a case of compound heterozygosity in the *CASQ2* (c.1074_1075delinsC and c.1176_1178delACAG) identified in a 7.3‐year‐old female (patient 4) with CPVT. We conclude that this novel frameshift variant of c.1175_1178delACAG in the last exon might have a mild effect on the protein and result in a very mild phenotype.

The c.838+1G>A in the downstream intron region of exon 8 is a newly discovered splice site variant in this study. The donor site score was predicted by the NNSPLICE 0.9 version (January 1997) of the splice site predictor (http://www.fruitfly.org/seq_tools/splice.html). The score was decreased, suggesting that the cleavage site variant had an effect on the encoded protein. A recent study has shown that it is possible to amplify *CASQ2* mRNA from platelets, enabling novel insights into the effects of splice site variants (Josephs, Patel, Janson, Montagna, & McDonald., 2017).

In contrast to the array of different variants, the phenotypes of the six Chinese children in the present study share many similarities: (a) all patients presented with a history of early onset exercise or emotion‐induced syncope. Several studies have demonstrated that a younger age at CPVT diagnosis is an independent predictor of future cardiac events (Hayashi et al., 2009). Patient 4 had the youngest age of onset and the most frequent syncope episodes. In Family 1, the onset age of patient 1 was 5 years, and syncope was more frequent and severe than in patients 2 and 3. (b) There was no obvious abnormality in the 12‐lead electrocardiogram at rest in the present CPVT patients, and QTc intervals were within normal parameters; Interestingly, patient 5 with homozygous variant of *CASQ2* (c.98G>A/p.R33Q) had relative resting bradycardia (54 bpm), which may be a direct effect of the impaired Ca^2+^ handling of his sinoatrial nodal cells, as a result of the effect of mutant calsequestrin protein. Holter and exercise test showed characteristic bVT and/or pVT, which is a key criterion for clinical diagnosis. (c) Delayed diagnosis of CPVT is common. Our patients had a mean delay of 3.4 years from first presentation to diagnosis. (d) Treatment with beta‐blockers had a favorable overall outcome: five patients were symptom‐free during the follow‐up period of 14–22 months. (e) However, the precise phenotype–genotype relationship in relation to *CASQ2* is unknown, and our results do not address this question. Further research in additional clinical cases should enable prediction of disease severity and allow better treatment outcomes for patients.

In conclusion, the present study is the first to examine *CASQ2*‐associated CPVT in young children in the Han Chinese population, with Holter and exercise tests showing characteristic bVT and/or pVT, which are key criteria for clinical diagnosis. Early treatment with beta‐blockers effectively improved prognosis. We identified three unreported variants in the *CASQ2* (c.1074_1075delinsC, c.1175_1178delACAG, and c.838+1G>A) that led to a rare autosomal recessive type of CPVT. Our findings expand the genotypic spectrum of this condition and provide a molecular basis for further studies of the mechanisms underlying *CASQ2*‐associated CPVT in Chinese patients.

## CONFLICT OF INTEREST

The authors declare no conflict of interest.
